# Advances in Diagnostic Imaging of Hepatopulmonary Syndrome

**DOI:** 10.3389/fmed.2021.817758

**Published:** 2022-01-10

**Authors:** Bi-Wei Luo, Zhi-Yong Du

**Affiliations:** Department of Hepatobiliary and Pancreas Surgery, Shenzhen People's Hospital (The Second Clinical Medical College, Jinan University; The First Affiliated Hospital, Southern University of Science and Technology), Shenzhen, China

**Keywords:** hepatopulmonary syndrome, diagnostic imaging, liver disease, IPVD, pulmonary vasodilation

## Abstract

Hepatopulmonary syndrome (HPS) is a serious pulmonary complication of progressive liver disease that leads to a poor clinical prognosis. Patients with HPS may develop acute respiratory failure, which requires intensive care and therapy. At present, the only effective treatment is liver transplantation; therefore, early diagnosis and timely treatment are of considerable significance. The three main features of HPS are liver disease, oxygenation disorder, and intrapulmonary vascular dilatation (IPVD). Diagnosing HPS is challenging due to the difficulty in detecting the presence or absence of IPVD. As such, imaging examination is very important for detecting IPVD. This paper reviews the imaging methods for diagnosing HPS such as ultrasound, dynamic pulmonary perfusion imaging, pulmonary angiography, and computed tomography.

## Introduction

When liver disease progresses to a certain stage, it is followed by the onset of pulmonary vasodilation, pulmonary gas exchange disorder, decrease in the arterial oxygen partial pressure, and clinical manifestations, which is called hepatopulmonary syndrome. In 1956, Rydell and Hoffbauer first reported a patient with liver cirrhosis complicated with a pulmonary arteriovenous fistula and described the pathophysiology of hypoxemia (1). In 1977, Kennedy and Knudson first used the term “hepatopulmonary syndrome (HPS)” to describe pulmonary vascular disease secondary to liver cirrhosis and it has been used thereafter (2). Subsequently, Sherlock further elaborated on hepatopulmonary syndrome (3). There is a clear connection between hypoxemia and liver disease. In presence of liver disease, it appears that pulmonary vasodilatation (PV) leads to a mismatch between ventilation/perfusion and diffusing capacity. The diagnostic criteria for HPS: (1) presence of liver disease, (2) evidence of intrapulmonary vascular dilatation (IPVD), which is also the basic pathological change, and (3) a pulmonary bubble arterial oxygen differential pressure [P [A-a] O_2_ gradient] that is ≥15 mmHg (or >20 mmHg if age ≥65 years). The severity of HPS can be classified according to the PaO_2_ into either mild (PaO_2_ ≥80 mmHg), moderate (PaO_2_ 60–79 mmHg), severe (PaO_2_ 50–59 mmHg), or very severe (PaO_2_ <50 mmHg) (4). HPS mostly occurs in patients with liver cirrhosis, with an incidence rate of 9.45–42.5% (5–10). HPS is a serious pulmonary complication of end-stage liver disease. The mortality of patients with HPS is significantly high and the prognosis is poor (10). Early detection and timely treatment may help improve the prognosis. HPS should be considered if there is abnormal pulmonary arterial oxygenation in the presence of liver disease; however, abnormal blood oxygen saturation alone is not enough to diagnose HPS (9). The focus of clinical diagnosis is to identify evidence of PV in patients with chronic liver disease complicated with hypoxemia. Therefore, clinical assessment must be combined with the results of imaging examination. This paper presents a comprehensive review of the latest developments in the diagnostic imaging of HPS.

## Progress in Ultrasound Use for the Diagnosis of HPS

With the onset of HPS, patients have a higher cardiac output and hyperdynamic circulation, resulting in corresponding changes in the cardiac structure. HPS can be indirectly diagnosed by measuring the cardiac structure using cardiac color Doppler ultrasound. Zamirian conducted a cardiac B-ultrasound examination on adult patients with confirmed HPS and found that the left atrial volume (LAV) was increased significantly. In patients with liver cirrhosis, HPS is considered if the measured LAV is >50 ml, and sensitivity and specificity are estimated to be 86.3 and 81.2%, respectively (11). This is mainly due to the right to left shunt in patients with HPS, resulting in increased cardiac output and increased left heart volume (11). A previous study proved that increased LAV can reflect the presence of HPS in patients with liver cirrhosis, and increased systemic blood velocity through the mitral valve can also indirectly indicate the presence of HPS (12). Moreover, Soulaidopoulos found that absolute left ventricular (LV) global longitudinal strain (GLS) values are significantly lower in patients with HPS compared to cirrhotic patients without HPS (13). Furthermore, results from a previous study suggest that systolic pulmonary artery pressure, right ventricle wall width, and early filling (E) and late filling (A) ratio (E/A ratio) can predict IPVD (14). The right ventricular diastolic dysfunction rate also increases significantly in patients with liver cirrhosis, especially in patients with HPS (15). As HPS can cause changes in the cardiac structure, cardiac ultrasound measurement can help in making an indirect diagnosis. This method is simple and convenient and especially suitable for preliminary screening of HPS in primary medical units (16).

## Progress in Contrast Enhanced Echocardiography (Cee) for the Diagnosis of HPS

The hand vibration method is used to produce microbubbles with a diameter ≤ 90 μm in normal saline, followed by injection into an upper limb vein of the patient. If the patient does not have a right to left shunt, the microbubbles will be captured and absorbed by the pulmonary capillary bed, and will not be able to reach the left cardiac system. Conversely, if the subject has pulmonary capillary dilation or a right to left shunt, the microbubbles will reach the left cardiac system, and can be detected in the left heart by ultrasonic examination. This method is called contrast-enhanced echocardiography. At present, the commonly used methods are contrast-enhanced transthoracic echocardiography (CTTE) and contrast-enhanced transesophageal echocardiography (CTEE). CTTE is a simple, non-invasive technique, low cost, and safe. However, CTEE is an invasive procedure that is relatively complex and entails a high cost. However, CTEE can provide excellent image quality, has a higher sensitivity that can improve the detection rate, and is significantly better for the diagnosis of PV compared to CTTE (17–19).

Some researchers have attempted to increase the sensitivity of CEE by changing the operation mode. For example, CEE examination in a standing position is observed to be better than that in a supine position, which may be due to the expansion of the pulmonary basal blood vessels and increased blood flow because of gravity (20, 21). Moreover, 3D CEE can obtain more 2D images and provide more data for analysis. CEE has a higher sensitivity and can better diagnose HPS compared to CTEE (22). A previous study estimated the erythrocyte pulmonary transit time (PTT) by detecting the time from the right atrium to the left atrium by CEE. It was found that PTT may be a useful parameter for evaluating arterial oxygenation in patients with chronic liver disease with early HPS (23). Another study also revealed that contrast enhanced transcranial Doppler was more sensitive for the detection of IPVD with a low false positive rate (24).

CEE is the most commonly used diagnostic imaging method for PV and the right to left shunt. It is simple, convenient, minimally invasive, and highly sensitive. At present, it is the gold standard for diagnosis. CEE also has some limitations. As an example, since many patients with liver cirrhosis may have normal blood gas content, the positive detection rate for such patients is only 40% by CEE. Further, other diseases of the lung can also lead to abnormal gas exchange. Therefore, using CEE, positive findings in the lungs alone are not enough for the diagnosis of HPS.

## Progress in Technetium 99m-Labeled Macroaggregated Albumin (Tc-MMA) Dynamic Lung Perfusion Scan for the Diagnosis of HPS

Dynamic pulmonary perfusion imaging is one of the common methods for diagnosing pulmonary vascular abnormalities in HPS. Technetium labeled human serum albumin polymer particle is a large albumin particle with a diameter >20 μm. A certain amount of Tc-MMA is injected into the peripheral vein of the patient at one time. The whole process of the radionuclide passing through cardiopulmonary vessels is recorded in the anterior and posterior positions by the γ camera during cardiovascular dynamic imaging. After 5 min, the posterior and anterior positions are taken for whole-body imaging, and the computer is used for synchronous data collection and imaging. The whole-body and bilateral lung radioactivity counts are calculated by the region of interest method, and the right to left shunt ratio of Tc-MMA is also calculated. Tc-MMA persists in the capillary beds with a diameter of 8–15 μm, and when the pulmonary capillaries show abnormal dilation and arteriovenous communication, Tc-MMA can pass through the abnormally dilated pulmonary vessels. This can help in obtaining extrapulmonary organ imaging and determining the intrapulmonary partial flow, which can help to diagnose patients with HPS (25, 26). Zhao et al. evaluated the sensitivity, specificity, and accuracy of the brain and systemic uptake of 99mTc-MAA in IPVD, and found that the whole-body uptake was better than simple brain uptake (27). The overall sensitivity of 99mTc-MAA in the diagnosis of HPS is 18.9% in all HPS cases, which is relatively low, but it is 66.7% in severe to very severe cases. Consequently, it can be used in patients with pulmonary disease and severe to very severe HPS (28).

Tc-MAA perfusion lung scan is more sensitive than CEE in detecting an intrapulmonary shunt in children with HPS (29). Some studies have suggested that there is no significant difference between the two in detecting an intrapulmonary shunt in children with HPS (6). In general, the detection sensitivity of 99mTc-MAA is lower than that of CEE, although its specificity is higher than that of CEE for the diagnosis of HPS. However, the greatest limitation of this imaging examination is that it cannot distinguish between an intracardiac and intrapulmonary shunt, nor can it evaluate cardiac function and pulmonary artery pressure. In addition, it can produce false positive results because non-bound technetium can pass through the vessels with a normal width.

## Progress in Pulmonary Angiography for the Diagnosis of HPS

Pulmonary angiography refers to selective pulmonary angiography carried out by puncture of peripheral blood vessels (usually femoral veins) and insertion of catheters. According to the different angiographic manifestations and pathophysiological characteristics, HPS can be divided into type I and type II (30). In type I, normal or diffuse, small, spider-like branching dilatations can be seen in the early stage. With the progression of dilatation, blotchy or spongy diffuse dilatation can be observed. Type II is relatively rare, showing anatomic arteriovenous malformations similar to spider nevus (31). Pulmonary angiography can directly reveal the dilated blood vessels and can also be used for embolization and other treatments (32). In addition, the determination of PTT by pulmonary angiography is very useful for the diagnosis of HPS, with higher sensitivity compared to the Tc-MAA lung perfusion scan, and may even be used to quantify the degree of intrapulmonary shunting (33).

Pulmonary angiography has not been widely used in clinical settings as it is invasive and expensive. It is mainly used in patients with persistent hypoxemia who have a poor response to inhaled pure oxygen and need intrapulmonary vascular embolization. In some HPS patients, PV is microscopic, and pulmonary angiography may not reveal any abnormality.

## Advances in the Computed Tomography (Ct) Diagnosis of HPS

The principal manifestations of chest CT in HPS include thickening and distortion of the lung basal texture, medium-sized nodular or reticular nodular shadows at the bottom of the lung, arteriovenous malformations, cardiac enlargement, and pulmonary artery widening (34). In addition to the number and location of pulmonary arteriovenous malformations, CT can also display the lung parenchyma pattern such as a mosaic pattern, which is characterized by alternating low attenuation areas (pulmonary vessels with reduced diameter) and high attenuation areas (pulmonary vessels with normal diameter). This mosaic pattern of the lung parenchyma is speculated to be associated with HPS and may be a potentially useful marker for the diagnosis of HPS, but still needs to be confirmed by large research studies (34).

High-resolution CT (HRCT) can show the expansion of the subpleural vessels that extend to the pleura, which is helpful for the diagnosis of HPS (35). Chest HRCT can also help in the diagnosis of HPS by calculating the ratio of the pulmonary artery to the bronchial diameter in the basal segment of the right lower lobe (36). Some studies have shown that although the arterial bronchial ratio of the basal segment in patients with liver disease is greater than that in the normal control group, this vasodilation is not serious in patients with HPS (37), and the incidence is low (7). CT cannot adequately distinguish if liver disease is complicated by HPS. For example, in a patient with HPS complicated with emphysema, an intrapulmonary shunt examination by 99mTc-MAA showed that the systemic shunt rate was 43% and there was no vasodilation on chest CT (38). Therefore, more research data are needed to support CT imaging for HPS.

In one study, lung images were obtained by single-photon emission computed tomography and CT. After merging, the images revealed that there were subpleural reticular nodular shadows and/or dilated blood vessels at the bottom of the lung in patients with HPS. This seems to be a characteristic CT manifestation of intrapulmonary arteriovenous communications in the lungs of HPS patients (39).

## Discussion

With the development of HPS in patients with liver disease, there are different degrees of abnormal dilatation of the pulmonary vessels. The rapid and accurate diagnosis of HPS through an imaging examination is particularly important. Routine cardiac B-ultrasound can indirectly diagnose HPS by measuring the changes in the ventricular structure. At present, contrast echocardiography and Tc-MAA dynamic lung perfusion imaging can indirectly indicate the existence of an intrapulmonary right to left shunt. It is the most used examination method in the clinic, with a high sensitivity but low specificity. Pulmonary angiography can directly show the changes in the pulmonary vessels with high sensitivity and specificity, but it is more traumatic, expensive, and limited in application. CT can directly show the morphological changes in the pulmonary vessels, and facilitate the diagnosis of HPS based on specific signs. Although there are still some controversies, it can help in the diagnosis of HPS. With the recent developments in multi-energy computed tomography (MECT) diagnostic technology, it has been used in the clinical diagnosis of various diseases of the whole body, especially vascular imaging. Researchers have applied MECT in the diagnosis of pulmonary vascular disease because it provides additional information for assessing the pulmonary vascular system (40). MECT provides new avenues for research and may become a new diagnostic method for HPS.

Overall, the progress of research on the diagnostic imaging of HPS has been relatively slow. In the future, there is a need to increase the clinical and basic research on HPS to further deepen the understanding of its pathogenesis and investigate new examination techniques to improve diagnostic efficiency.

## Author Contributions

B-WL: writing—original draft. Z-YD: writing—review and editing and funding acquisition. All authors contributed to the article and approved the submitted version.

## Funding

This work was supported by Shenzhen Key Medical Discipline Construction Fund (No. SZXK015) and Science and Technology Innovation Foundation of Shenzhen (JCYJ20190807144807510).

## Conflict of Interest

The authors declare that the research was conducted in the absence of any commercial or financial relationships that could be construed as a potential conflict of interest.

## Publisher's Note

All claims expressed in this article are solely those of the authors and do not necessarily represent those of their affiliated organizations, or those of the publisher, the editors and the reviewers. Any product that may be evaluated in this article, or claim that may be made by its manufacturer, is not guaranteed or endorsed by the publisher.
